# Q fever and early pregnancy failure: a Scottish case–control study

**DOI:** 10.1530/RAF-21-0072

**Published:** 2021-12-24

**Authors:** Nick Wheelhouse, Sadie Kemp, Jo E B Halliday, Efstathios Alexandros Tingas, W Colin Duncan, Andrew W Horne

**Affiliations:** 1 School of Applied Sciences, Edinburgh Napier University, Sighthill Court, Edinburgh, UK; 2 Institute of Biodiversity, Animal Health and Comparative Medicine, College of Medical Veterinary and Life Sciences, University of Glasgow, Glasgow, UK; 3 School of Engineering & the Built Environment, Edinburgh Napier University, Merchiston Campus, Edinburgh, UK; 4 MRC Centre for Reproductive Health, Queen's Medical Research Institute, The University of Edinburgh, Edinburgh Bioquarter, Edinburgh, UK

## Abstract

Q fever is a bacterial disease that passes between animals and humans and causes disease in both. The disease has been associated with pregnancy complications including miscarriage. This study was undertaken to identify if Q fever exposure was correlated with miscarriage in 369 women attending a pregnancy support unit in Edinburgh. The women in the study were in two groups, the miscarriage group with 251 women who had experienced a miscarriage and a control group of 118 women who had not experienced miscarriage. Three women were found to be positive for Q fever antibodies, suggesting that they had previously been exposed to the infection and all of them were from the group who had experienced miscarriage. The study indicates that Q fever is relatively rare in women attending an urban Scottish hospital suggesting that the infection is not a major cause of miscarriage in this population. However, as Q fever antibodies could only be found in women within the miscarriage group, it suggests that the infection cannot be ruled out as a potential cause of miscarriage in individual cases.

Q fever is a zoonotic disease with a worldwide distribution caused by the bacterium *Coxiella burnetii* (*Cb*). The infection is primarily known as a clinical cause of pregnancy loss in livestock. *Cb* has also been linked to reproductive complications in humans, potentially as a result of damage to the placenta caused directly by bacterial colonisation or indirectly via the host immune response to infection (Ghanem-Zoubi & Paul 2020).

Within the UK, clinical cases of Q fever are considered a relatively rare occurrence (Halsby *et al*. 2017) but most cases of Q fever present with self-limiting flu-like symptoms and remain largely undiagnosed. One UK study suggested that exposure was endemic (mean prevalence in a random sample of participants in a population-based survey 12.8%) and found a significant correlation between Q fever seropositivity and history of adverse pregnancy outcomes in women (McCaughey *et al*. 2008). A second study identified a mean prevalence of 4.6% in pregnant women attending a clinic in London (Baud *et al*. 2009) suggesting variation in exposure levels between different demographic groups and also potentially regionally. Due to the relative paucity of data on Q fever seroprevalence across the UK and the potential link with adverse pregnancy outcomes, we investigated the prevalence of Q fever and the risk of miscarriage in a population of Scottish women.

We analysed the serum from 369 participants. Clinical characteristics and recruitment criteria have been previously described (Horne *et al*. 2020). Briefly, we recruited women with ultrasonography confirming the absence of a fetal heart in the first trimester of pregnancy (miscarriage group) and women with normal pregnancies that had progressed into the third trimester (control group) from the same catchment population. The Scotland A Research Ethics Committee approved this study and written informed consent was obtained from all participants. Serum samples were analysed for the presence of specific phase I and phase II anti-*Coxiella burnetii* IgG by commercial ELISA (Serion Diagnostics, Würzburg, Germany) using the manufacturer’s recommended cut-offs to determine the positivity.

In the study, 3/369 (0.8%) participants were found to be seropositive for *Cb* IgG; 2 were positive for phase I IgG and 1 was positive for phase II IgG (Table 1). All seropositive women were identified from the group of women who had suffered from a miscarriage (3/251 participants; 1.2%) with no seropositive individuals identified within the control group (0/118 participants).

**Table 1:** Summary of participant characteristics. Data are presented as *n* (%) or as indicated.

	Miscarriage	Control	Total	*P*-value*
*n*	251	118	369	
Age in years, median (95% CI)	34 (32–35)	33 (32–35)		0.72
BMI, mean (95% CI)	26.0 (25.0–27.0)	25.6 (24.9–26.3)		0.55
Prior miscarriage	106 (41.4)	NA		NA
Prior live births	127 (50.6)	85 (72.0)		<0.001
*Coxiella burnetii* serostatus				
Total IgG	3 (1.20)	0	3 (0.81)	0.55
Phase I IgG	2 (0.80)	0	2 (0.54)	
Phase II IgG	1 (0.40)	0	1 (0.27)	

*P < 0.05 indicates statistical significance.

These findings indicate that exposure to *Cb* is rare in this cohort of patients attending an urban Scottish hospital unit, suggesting that Q fever is unlikely to be a major contributory cause of medically diagnosed first-trimester miscarriage in this population. However, the presence of seropositive individuals within the miscarriage group suggests that infection with *Cb* cannot be dismissed as a potential contributory cause. Phase I seroconversion in two of the women is also of concern. While many studies analyse phase II antibodies which are an indicator of a present or relatively recent infection, phase I seroconversion is suggestive of chronic Q fever which is a relatively rare condition (less than 5% of cases) but can lead to significant long-term health consequences. These findings indicate the need for further investigation of the role of *Cb* infection as a cause of pregnancy loss in the UK, with a potential focus on at-risk groups, including those working in rural professions where the risk of exposure is significantly greater (Halsby *et al*. 2017).

## Declaration of interest

Andrew Horne is a Co-Editor-in-Chief and Nick Wheelhouse is an Associate Editor of Reproduction and Fertility. Andrew Horne and Nick Wheelhouse were not involved in the review or editorial process for this paper, on which they are listed as authors. The other authors have nothing to disclose.

## Funding

This work was made possible by the funding from Tommy’s Baby Charity, the Medical Research Council (MR/N022556/1) and through an Edinburgh Napier Strategic Research & Knowledge Exchange Fund grant (2748206).

## Author contribution statement

N W and A W H conceived, supervised the study and co-wrote the manuscript. S K performed the experiments. J H, W C D and E A T for help with the statistical and intellectual interpretation of the data and provided critical revision of the manuscript.
